# Fabrication approaches for the creation of physical models from microscopy data

**DOI:** 10.1186/s41205-017-0011-6

**Published:** 2017-02-14

**Authors:** Benjamin L. Cox, Nathan Schumacher, John Konieczny, Issac Reifschneider, Thomas R. Mackie, Marisa S. Otegui, Kevin W. Eliceiri

**Affiliations:** 1 Department of Medical Physics, University of Wisconsin-Madison, 1111 Highland Ave, Madison, WI 53705 USA; 2 Medical Engineering Group, Morgridge Institute for Research, 330 N Orchard St, Madison, WI 53715 USA; 3 Laboratory for Optical and Computational Instrumentation, University of Wisconsin – Madison, 1675 Observatory Drive, Madison, WI 53706 USA; 4 Rapid Prototyping Center, Milwaukee School of Engineering, 1025 North Broadway Street, Milwaukee, WI 53202 USA; 5 Department of Botany, University of Wisconsin-Madison, 430 Lincoln Drive, Madison, WI 53706 USA

**Keywords:** 3D-printing, Microscopy, 3D-imaging, Instructional models, 3D-visualization

## Abstract

**Background:**

Three-dimensional (3D) printing has become a useful method of fabrication for many clinical applications. It is also a technique that is becoming increasingly accessible, as the price of the necessary tools and supplies decline. One emerging, and unreported, application for 3D printing is to aid in the visualization of 3D imaging data by creating physical models of select structures of interest.

**Methods:**

Presented here are three physical models that were fabricated from three different 3D microscopy datasets. Different methods of fabrication and imaging techniques were used in each case.

**Results:**

Each model is presented in detail. This includes the imaging modality used to capture the raw data, the software used to create any computer models and the 3D printing tools used to create each model. Despite the differences in their creation, these examples follow a simple common workflow that is also detailed.

**Conclusions:**

Following these approaches, one can easily make 3D printed models from 3D microscopy datasets utilizing off the shelf commercially available software and hardware.

## Background

Although 3D printing is now widely used for medical applications [1–3], there are lesser-known biomedical applications, including the manufacturing of complex parts for research, such as microfluidic components, and for education [4, 5]. Aiding both research and education, 3D printing can be used to create physical models from biomedical imaging data. Physical 3D models are useful as they allow researchers to hold and feel a structure that they might otherwise only be able to see on a computer screen [6, 7]. These models can prove especially useful in fields where concepts are often difficult to spatially comprehend from a two-dimension (2D) image. For these reasons, these models are also extremely effective teaching tools to help students understand complex 3D biological phenomena, such as membrane architecture and dynamics.

Most 3D printing of biomedical images to date has utilized clinical imaging methods such as computed tomography (CT), positron emission tomography (PET), magnetic resonance imaging (MRI) and 3D ultrasound [1, 2, 8, 9]. Here we describe 3D printing from optical imaging methods, such as confocal microscopy and multiphoton microscopy. The creation of the models, discussed here, highlight similarities that can be applied to create models from many microscopy techniques including electron microscopy and other optical modalities.

**Fig. 1:**
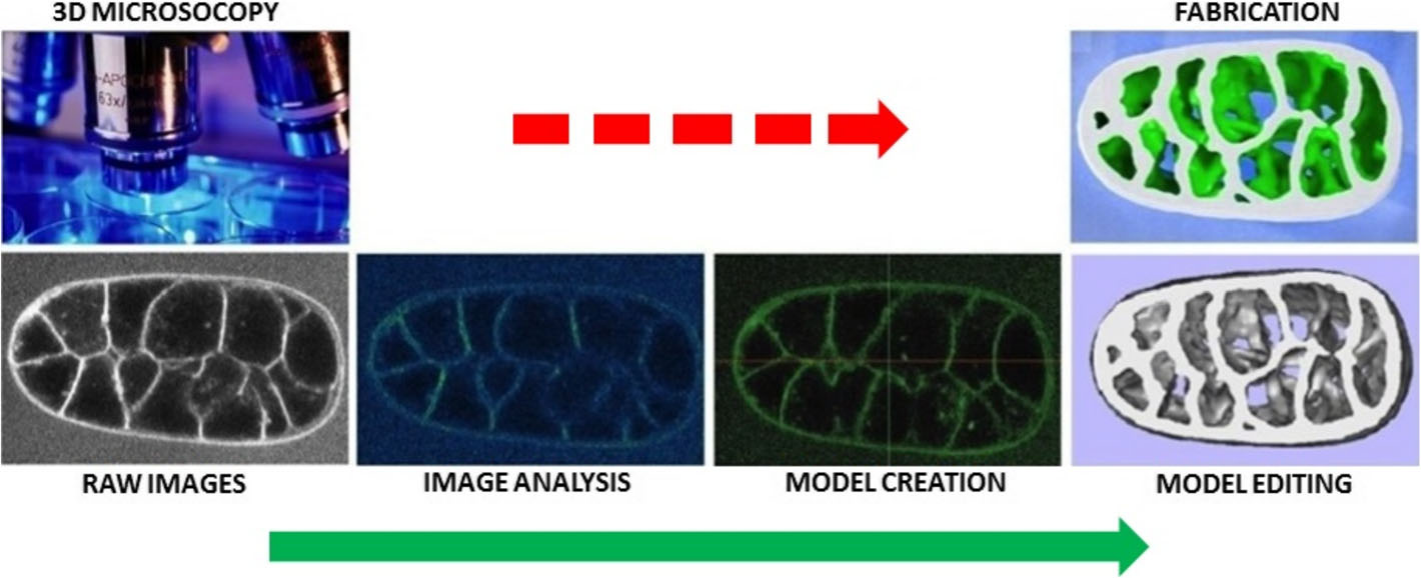
This is a schematic depicting a typical workflow from imaging to physical part. A sample is imaged. The raw images need to be analyzed and a model needs to be created from them. The model can be edited using some of the software described in this paper before a final part is fabricated

3D printing is similar to 3D optical imaging techniques in several ways. Just as in optical imaging, where compiling 2D images into a stack creates a 3D dataset; compiling 2D slices into the final printed object creates a 3D part. The workflow from image data to the creation of a final model is fairly straightforward in principle. Following the acquisition of the image data, a generation of the corresponding 3D computer model is required, and then the appropriate 3D printing method must be selected to finally create the physical model (Fig. 1). While straightforward, this process is relatively un-documented. An aim of this paper is to document this process, reducing the expertise required to make physical models from different types of 3D image datasets.

There are an increasing number of free and open source software packages that are capable of creating a computer model from 3D image data as well as affordable and fairly high resolution 3D printers that are on the market or slated to be released soon. In addition, there are more choices in printing materials, depending on the needs for resolution, cost and durability. These developments have combined to make this process more accessible and practical than it has ever been [10]. Presented here are three physical models derived from different optical imaging modalities that were created using different fabrication techniques. These image models were chosen for their biological relevance and ability to represent different image modalities and fabrication challenges. Alternate options for fabrication and file creation and the implications of the increased accessibility of this process are also discussed.

## Methods

### General pipeline for 3D printing of 3D microscopy datasets

The tools required to fabricate models from 3D microscopy datasets fall into three categories. First, there are the imaging tools that are required to collect the raw data. Second, there are a variety of different software tools that are needed to convert the raw data into usable file formats and then to create the 3D computer models used for printing. Finally, there are the 3D printing tools needed to create the final models. The specific tools used in the creation of each model highlighted here are summarized below and in Table 1.

**Table 1:** Tools used for model creation

Model	C. Elegans embryo	Distal tip cell	Prevacuolar compartment
Imaging			
Imaging Modality	Multiphoton Microscopy	Confocal Microscopy	Electron Tomography
Imaging equipment	Femtosecond-Pulsed 1047 nm Nd:YLF Laser	Zeiss LSM510 Laser Scanning Confocal Microscope	Tecnai F30 Transmission Electron Microscope
Software			
TIFF Stack Creation	Fiji (fiji.sc)	Fiji (fiji.sc)	N/A
STL File Creation	Mimics (Materialise)	Mimics (Materialise)	IMOD and MeshLab (meshlab.sourceforge.net/)
Model Editing	FreeForm Modeling, now Geomagics (3D Systems)	Magics (Materialise)	MeshLab (meshlab.sourceforge.net/) and Magics (Materialise)
Printing Software	Zprint Software (3D Systems – Previously Zcorporation)	3D Lightyear (3D Systems)	CatalystEX (Stratasys)
3D Printing			
3D Printing Equipment	Spectrum Z510 (3D Systems – Previously Zcorporation)	Viper Si2 SL Machine (3D Systems)	Dimension Elite FDM Printer (Stratasys)
3D Printing Material	Plaster Powder	Accura60 (3D Systems)	ABS*plus* (Stratasys)
Other Materials	N/A	N/A	Acrylic Resin

### Imaging

Any microscopy imaging modality that creates 3D datasets can be used for the creation of physical models. This includes microscopy techniques like confocal microscopy [11] and multiphoton microscopy [12] that section tissue by taking 2D images at different depths through the sample, as well as optical techniques that are tomographic, like optical projection tomography (OPT) [13] and electron tomography [14] that use 2D projections at many different angles to create a 3D dataset. The imaging modalities used to collect the three independent datasets for the models described here are confocal microscopy [11], electron tomography [14] and multiphoton microscopy [15].

Specifically, a *Caenorhabditis elegans* embryo expressing a membrane-localized green fluorescent protein (GFP) marker was imaged using multiphoton laser scanning microscopy [16]. A *C. elegans* distal tip cell expressing a GFP marker was imaged with 63X 1.4NA objective and 488 nm excitation on a Zeiss LSM510 laser scanning confocal microscope. And a prevacuolar compartment of a high-pressure frozen/freeze substituted maize aleurone cell was imaged by dual electron tomography in a Tecnai F30 transmission electron microscope [17].

### Software

A combination of different software packages were used to generate 3D models for fabrication, including both commercial and open-source software. For the creation of physical models from imaging data, the raw data must first be converted to an acceptable file format. Commercial microscopes often use proprietary file types when writing data files. Fiji [18], an open source image viewing and editing software, is adept at handling these types of commercial file types, using its Bio-Formats Library [19], creating TIFF stacks from raw data that can be easily handled by most software packages.

Once the data was in TIFF format, a variety of software packages were used for different steps of the model creation process. Mimics (Materialise, NV, Leuven, Belgium) was used to visualize imaging data and convert files into standard tessellation language (STL) format, the format used for 3D printing, Solidworks (Dassault Systemes, France) was used to view STL files and assess their quality, Magics (Materialise) was used to modify individual surfaces in STL files and Geomagics (3D Systems) was used for surface for smoothing. The *C. elegans* embryo model, color details were added to the model using Zprint software (Zcorporation, now 3D Systems). The dual axis tomogram of a plant prevacuolar compartment was calculated using the IMOD package [20].

It is important to note that the modifications performed using software packages like Magics (Materialise), noted above, may result in the loss of some of the quantitative information present in the original imaging data. However, these modifications are necessary to fix features that are difficult or impossible to print using a particular 3D printing technique. This might include things like floating features or very fine points.

### 3D printing

There are several different methods of 3D printing. Common to each method, however, is the creation of a 3D model from 2D slices. These methods differ only in how each process creates the individual slices. The types of 3D printing used to create the models described here are stereolithography (SL) [21] for the distal tip cell model, fused-deposition modeling (FDM) [22] for the mold and internal components of the prevacuolar model and powder-based inkjet 3D printing for the *C. elegans* embryo model. SL creates parts by curing photopolymers with an ultraviolet (UV) laser, FDM creates parts by extruding plastic cord to create a thin filament that is layered and powder-based inkjet 3D printing creates parts by applying a binder to layers of powder. Inkjet 3D printing can create full color parts by applying ink to the binder it uses.

The specific models of 3D printers that were used to fabricate the 3D models, include the Viper Si2 SL machine (3D Systems), the Spectrum Z510 machine (Zcorporation, now 3D Systems) and the Dimension Elite printer (Stratasys). 3D printing materials included, Accura60 (3D Systems) resin for the ViperSi2 system, a plaster powder (Zcorporation, now 3D Systems) for the Spectrum Z510 system and ABS*plus* corded plastic (Stratasys) for the Dimension Elite system.

As noted, the prevacuolar model was created using a molding process. The mold itself and several of the internal structures were printed using FDM out of ABS*plus*, but the model itself was not printed, but cast out of acrylic resin. The internal structures were placed inside the mold during casting. Also, the distal tip cell model was created using SL, but the model itself is burned inside a block of cured Accura60. This was done by overlaying dozens of copies of the computer model for the imaging data within a computer model of a surrounding block. During fabrication, the laser in the 3D printer dwelled over the areas of the block with the distal tip cell much longer, which overcured the resin, producing the brown color seen in the model.

## Results

Three physical models were generated from three distinct sets of 3D imaging data. These include a multiphoton microscopy model of a *C. elegans* embryo [16], a confocal microscopy model of the distal tip cell of *C. elegans,* and an electron tomography model of a prevacuolar compartment in a maize endosperm cell [17].

**Fig. 2:**
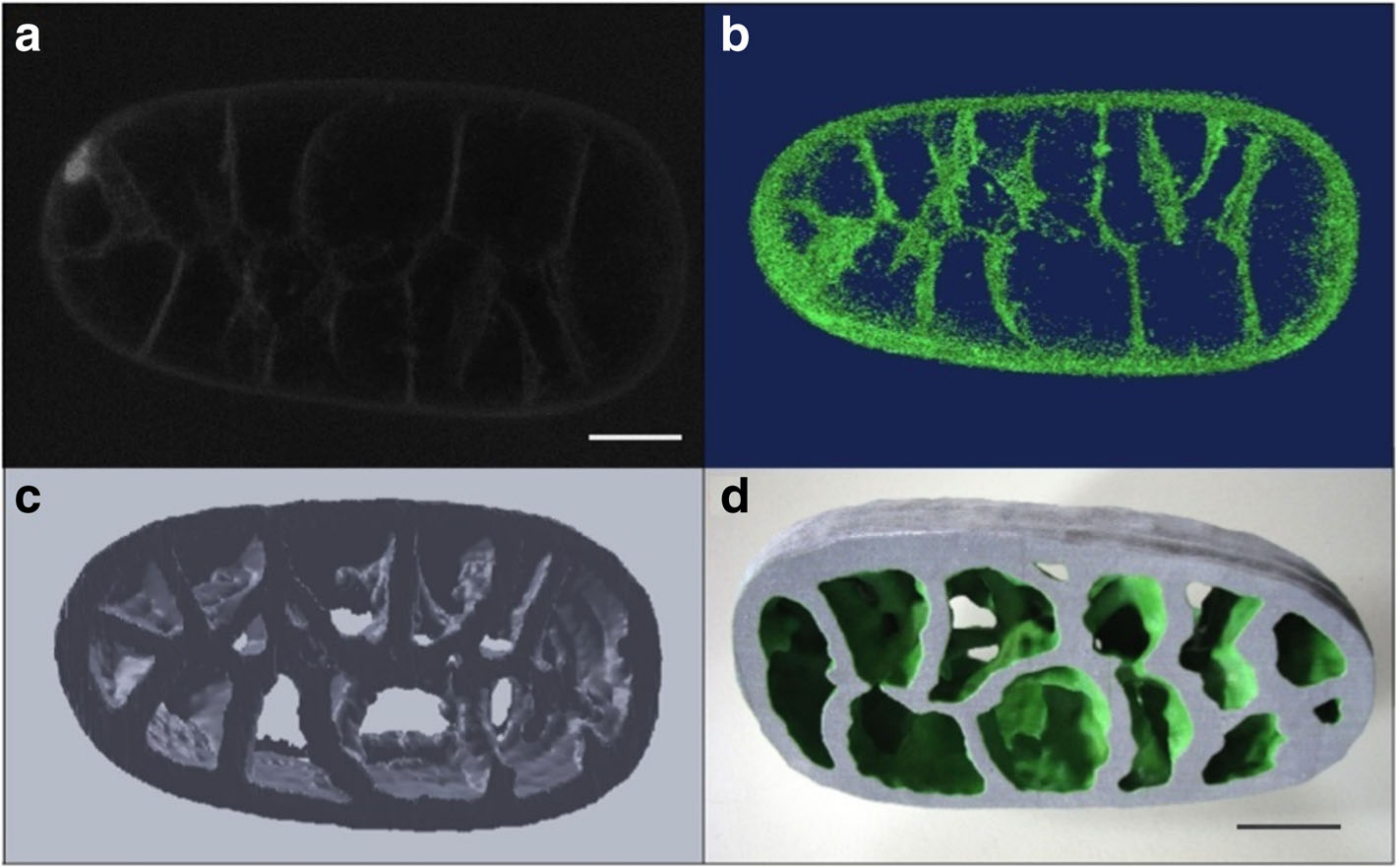
Images representing various steps in the creation of the *C. elegans* embryo model. **a** Original imaging data of a *C. Elegans* embryo. **b** Screenshot of the embryo after a threshold has been applied in Mimics. **c** Screenshot of the STL file of the thickened embryo model opened in SolidWorks. **d** Final printed model. The scale bar in Panel **a** is roughly 10 microns and the scale bar in Panel **d** is roughly 3 cm. The scaling factor of the model is roughly 3000:1

A *C. elegans* embryo with a membrane-localized GFP marker was imaged using multiphoton laser scanning microscopy to create a stack of images going through the sample [16]. After file conversion, the image stack was imported into Mimics, to generate a 3D computer rendering of the embryo. In Mimics, thresholding was performed as well as other operations to increase image contrast. Next, the STL file was imported to the FreeForm Modeling system for smoothing. This software is now commercially available from 3D Systems with the name Geomagics. The thickness of the surfaces was increased so that the thinner parts of the model could be printed. The final model was fabricated using a Spectrum Z510 machine, a machine that uses the inkjet 3D printing process (Fig. 2a-d). Color details were added to the model prior to printing using Zprint software (Zcorporation, now 3D Systems).

**Fig. 3:**
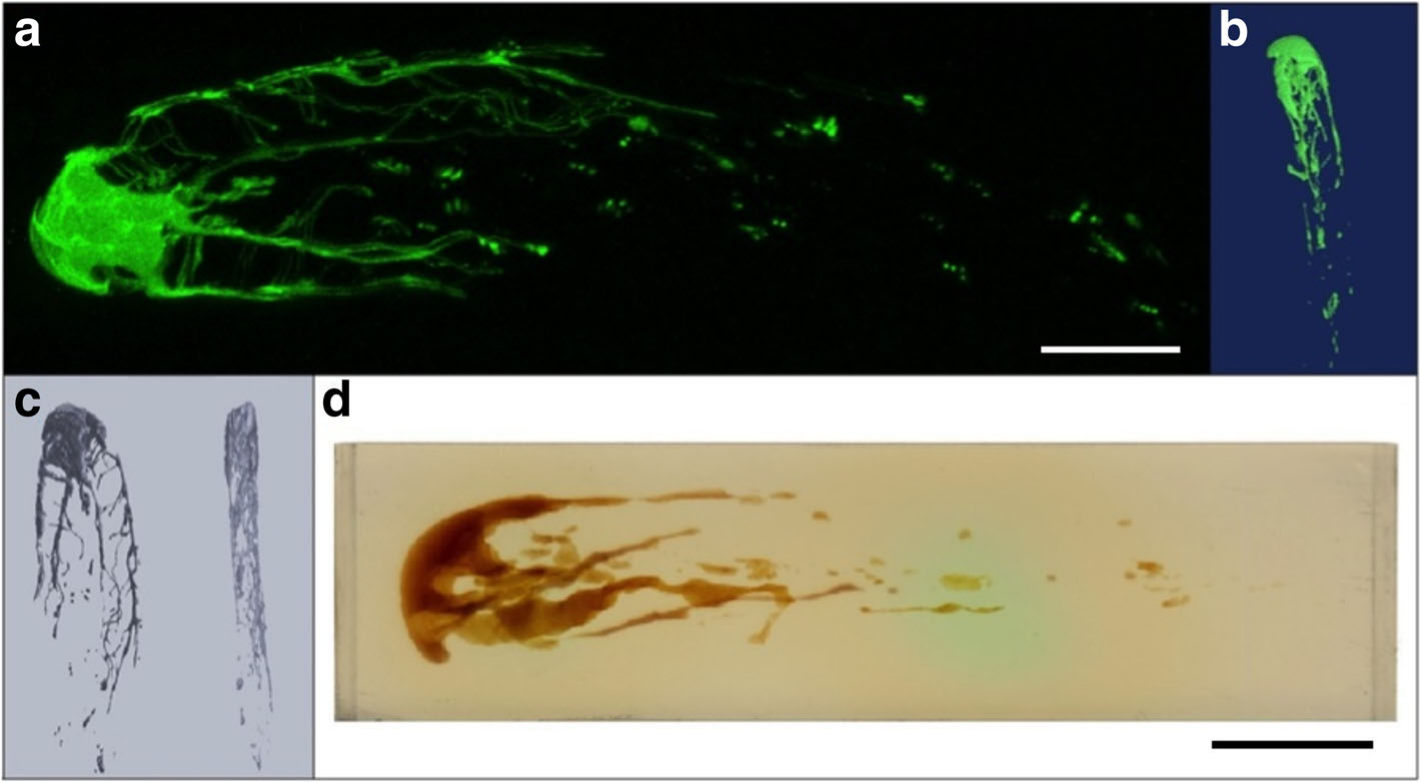
Images representing various steps in the creation of the distal tip cell model. **a** Original imaging data of a *C. elegans* distal tip cell. **b** Screenshot of the distal tip cell after a threshold has been applied in Mimics. **c** Screenshot of the STL file for the distal tip cell model opened in SolidWorks. **d** Final printed model. The scalebar in Panel **a** is about 5 microns and the scalebar in Panel **d** is about 2 cm. The scaling factor of the model is roughly 4000:1

Another model created using these processes is that of a somatic distal tip cell of *C. elegans,* a single cell with long extensions located at the distal end of the adult gonad. It forms part of the cellular microenvironment for the germ cells and is believed to control their maturation and release [23]. A distal tip cell expressing a GFP marker was imaged with a Zeiss LSM510 laser scanning confocal microscope. The raw image data was then imported into Mimics, where a threshold was applied and a virtual model was generated. This model was 3D printed using a Viper Si2 SL machine (3D Systems). It appears brown in the block of cured material, because several dozen copies of it were overlaid on top of one another, as described above. This caused the laser to dwell over these parts of the model longer, burning the material and creating the 3D model of the distal tip cell within a surrounding block of material (Fig. 3a-d).

**Fig. 4:**
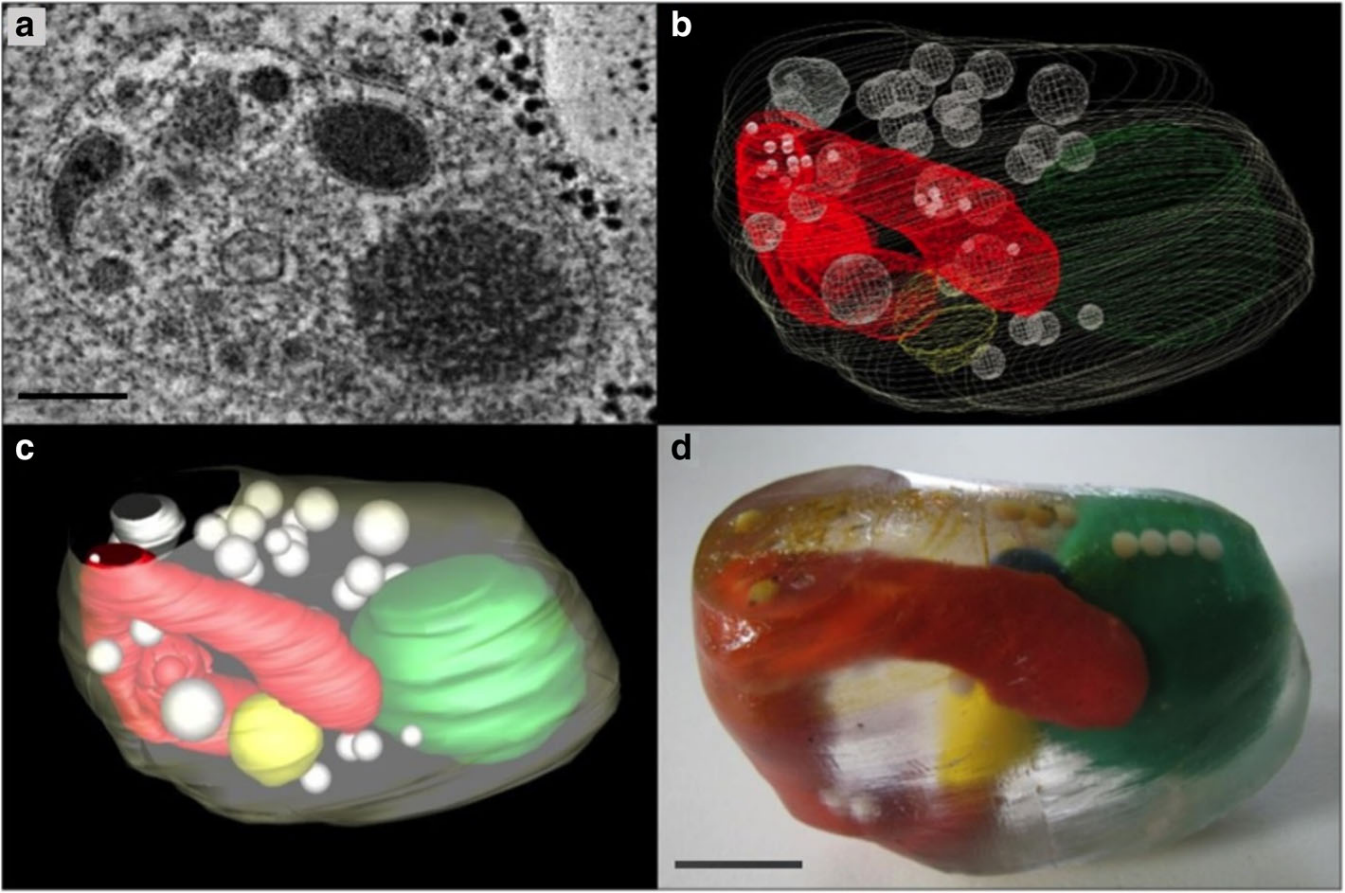
Images representing various steps in the creation of the prevacuolar compartment model. **a** Tomographic slice of a maize cell showing a prevacuolar compartment. **b** Screenshot of the segmented prevacuolar compartment as a mesh representation, with all of the interior components shown in different colors. Countours were traced in IMOD and the mesh model was created in MeshLabs. **c** Screenshot of a solid rendering of the mesh model created in MeshLabs. **d** Final model after casting the 3D printed components in acrylic using the 3D printed mold. The scalebar in Panel **a** is about 100 nm and the scalebar in Panel **d** is about 3 cm. The scaling factor of the model is roughly 300000:1

Lastly, a prevacuolar compartment of a maize plant cell was created. These organelles carry proteins and endoplasmic reticulum membranes to vacuoles during endosperm development [17]. A prevacuolar organelle was segmented from a two-dual axis electron tomogram of a maize endosperm cell using IMOD, an open source 3D reconstruction software. Using MeshLab, another open source program, this data was imported as a point cloud, which was then converted into a mesh to create surfaces, from which individual structures could be selected. Internal structures, such as protein aggregates and membranes were selected and rendered as surfaces of different colors. To fabricate this model, the internal components of the prevacuolar compartment were printed using a Dimension Elite printer (Stratasys) with different colors of ABS*plus* plastic. Also, a mold of the exterior of the compartment was printed using the Dimension Elite printer. The model for this mold was created using Magics (Materialise), by subtracting the prevacuolar compartment model from a larger block to form the outer structure of the mold. Acrylic resin was poured into the mold to form the model. The interior components of the prevacuolar compartment were added in steps as the mold was filled, so that they were as close to their correct biological position as possible (Fig. 4a-d).

## Discussion

The examples of models presented here all are vast improvements to 2D pictures when trying to convey complex biological concepts. However, none of these models are perfect. 3D printing is a continually improving technology. As more individual techniques to 3D print objects are developed and as existing techniques are refined, the quality of models produced will continue to improve.

There are specific improvements to existing 3D printing techniques that will benefit the generation of physical models like the ones presented here. A printer capable of printing different colors within a clear plastic material would make a model of the prevacuolar compartment more accurate. Also, the capability to produce models of objects in free space would make some of these models more useful. This is especially true in the case of the prevacuolar compartment and of the distal tip cell. Finally, as the feature resolution and strength of available materials advance, the overall quality of the models will improve.

The future of 3D printing and microcopy has a lot of potential and room for growth. Despite the success of the 3D printed models shown here, the technology, both in model creation and in model fabrication, still can struggle to match the spatial and temporal resolution of a dynamic 3D dataset. In this presented work we chose to work mostly with relatively straightforward 3D datasets. But many investigators want to show multiple models of a process changing over time for research and educational purposes. It can be costly to make the multiple models needed to capture an entire process and difficult to represent fine or floating details in intricate processes. 3D datasets that do not have clear spatial boundaries can also be difficult to reproduce faithfully.

The 3D electron tomography model of the prevacuolar compartment presented in this paper nicely illustrates these issues. In this case, the data on screen can sometimes better show the features of interest as you can see the components more clearly as you rotate virtually, while some of that perspective is lost in the printed model. This is especially true when examining processes that happen over time, such as protein trafficking in this case, where the components are more in free space and can get left out of a printed model. And if included, they would only represent one snapshot of a process that is dynamic. As more 3D modeling, 3D printing and materials options become available, these types of models will improve.

Another challenge is workflows and the accessibility of these tools. The process used here required commercial tools that are available in most 3D printing facilities but not necessarily user friendly or available to the benchtop biologist. It would be ideal if image processing programs, such as the popular image processing program ImageJ/FIJI [18], had better support for creating 3D model files like STL files directly. If the same programs that microscopy professionals use to process their data could also be used directly to ready their data for 3D printing, there would be more widespread use of these modeling techniques, as the cost and difficulties in workflow would be reduced.

The major uses of 3D printing of image models are for research purposes and for informal and formal science education. It is clear that a researcher would likely value having a physical representation of their dataset and that such a model might aid in discussion and informal science outreach. Such models, even if they do not lead directly to new insights, might facilitate others to become interested or be powerful demonstration aids for illustrating biological concepts. However, the path is less clear towards using such models for formal science education. A pedagogical study would need to be done to see if such printed models have utility in the classroom to teach fundamental concepts.

There have been informal classroom studies showing the utilities of using real research data in the classroom [24, 25]. Additionally, there have been published studies showing the advantages of 3D molecular models in the classroom [7]. However, to our knowledge, there have been no studies showing if 3D printed models are an effective aid in formal education. For example, in a developmental biology class studying embryonic development, would 3D printed models of developing embryos directly improve comprehension of core concepts?

Despite the described challenges in technology, adoption and education use, 3D printing of biological data does have great promise. These types of models offer a tangible method to visualize complex data. These examples and the process outlined here can serve as a guide to future studies of 3D printing of biological imaging data and will hopefully help further its application.

## Conclusions

3D printing technologies are being used for medical applications, but largely with medical imaging data from CT, MRI or PET. However, another biomedical application of 3D printing is the fabrication of models derived from optical imaging data. Models of microscopy datasets can aid both researchers and students in understanding complex biological problems. Three such models, each derived from a different imaging modality and fabricated with a different printing technology, are presented along with the details of their creation. A general workflow for the creation of other models of this type is also included. The emergence of free and open-source software tools, the reduction in price of 3D printing hardware and the creation of new 3D printing materials are all factors that continually increase the accessibility of these technologies. As that accessibility continues to increase, the models and methods outlined here can serve as a general guide to researchers and educators who want to make similar models.

## Abbreviations

2D: Two-dimensional
3D: Three-dimensional
CT: Computed tomography
FDM: Fused-deposition modeling
GFP: Green fluorescent protein
MRI: Magnetic resonance imaging
OPT: Optical projection tomography
PET: Positron emission tomography
SL: Stereolithography
STL: Standard tessellation language
UV: Ultraviolet
